# Editorial: (Ir)Relevance in education: individuals as navigators of dynamic information landscapes

**DOI:** 10.3389/fpsyg.2024.1546881

**Published:** 2025-01-14

**Authors:** Katarzyna Bobrowicz, Jean-Pierre Thibaut, Samuel Greiff, Margarita Pavlova

**Affiliations:** ^1^Department of Behavioural and Cognitive Sciences, University of Luxembourg, Esch-sur-Alzette, Luxembourg; ^2^Laboratoire d'Étude de l'Apprentissage et du Développement, CNRS UMR 5022, Université de Bourgogne, Dijon, France; ^3^School of Social Sciences and Technology, Technical University Munich, Germany; ^4^Department of Cognitive Science and Psychology, New Bulgarian University, Sofia, Bulgaria

**Keywords:** relevance, education, learning, digitalization, student

## 1 Background and aims

Scanning, selecting, and evaluating dynamically changing information landscapes for relevant information is a quotidian aspect of individual lives in the 21st century (Bobrowicz et al., 2022; Bobrowicz, 2024). Open and universal access to information as well as the increasing digitalization of societies, labor markets, and states place great demands on individuals' ability to establish whether given information can be trusted, is reliable, and relevant to their goals (Leu et al., 2017). While information truthfulness and reliability have been increasingly discussed in this context, the question whether education supports individuals in determining, evaluating, and acting upon the relevance of available information has remained somewhat overlooked in recent years. Given that such judgments are critical to learning, academic achievement, and job performance, the present Research Topic aimed, first and foremost, to promote a closer look at the role, the context, and the support of individual relevance judgments in psychological and educational research. Furthermore, the present Research Topic aimed to forge a connection between research on goal-oriented use of information and educational practice across the lifespan by inspiring experts to reflect on how individuals determine, apply, and evaluate information relevance, and are supported in all these activities by different practices of learning and instruction.

## 2 The concept of information relevance

**Figure 1:**
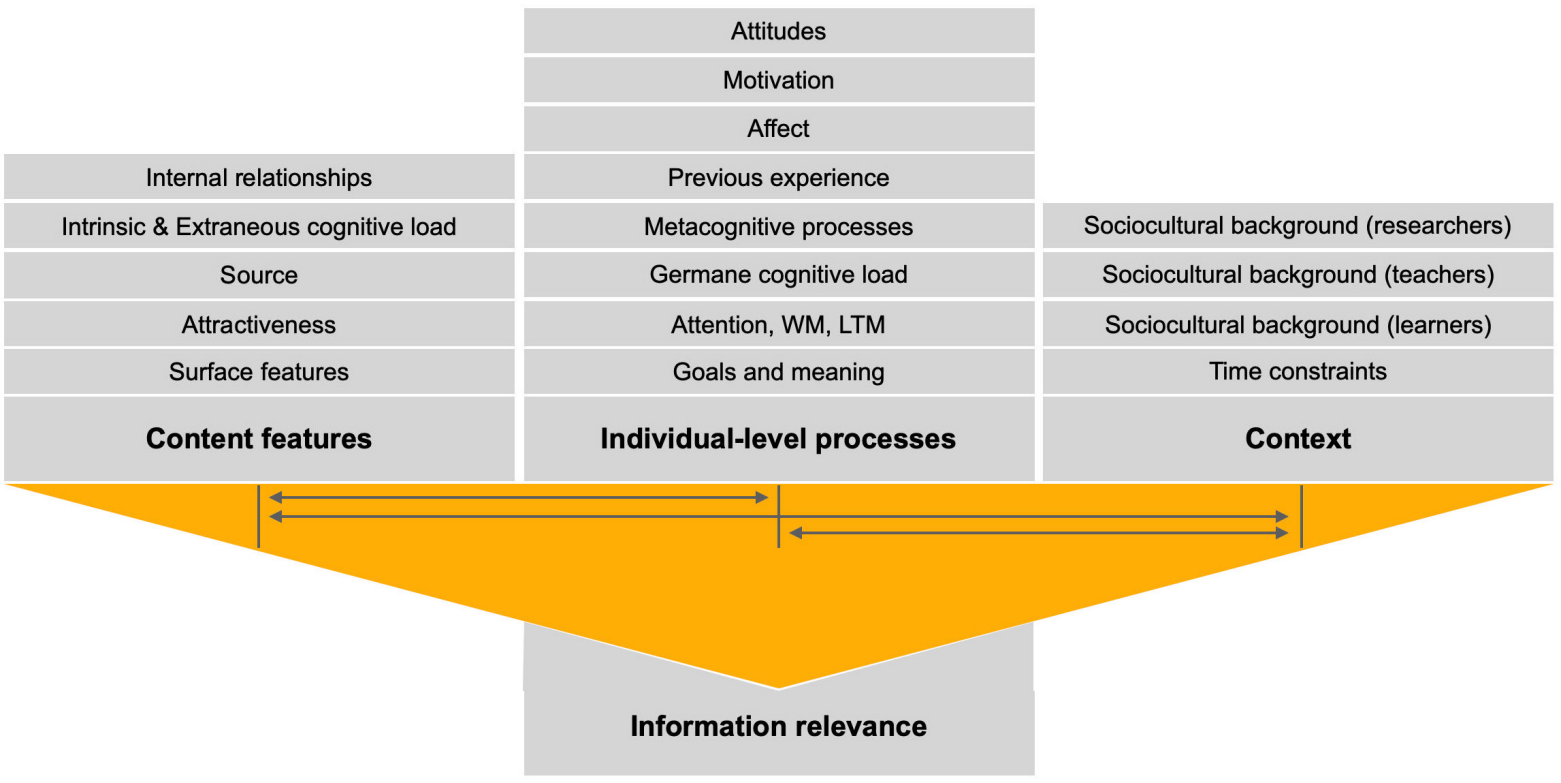
An overview of a single instance of information relevance. Of note, the content features, the individual-level processes, and the context continuously interact with one another during each instance of information relevance. WM, working memory; LTM, long-term memory.

Relevance is an intuitive yet elusive feature of information. It changes over time, in relation to previous decision-making, and in response to the internal states and external circumstances. In other words, information relevance is an ever-changing product of content features, individual-level processes (cognitive, affective, motivational), and the context (see Figure 1). The *content features* involve (1) surface features, such as color or shape, (2) visual/auditory/tactile attractiveness, (3) information source, (4) intrinsic and extraneous cognitive load (i.e., the load induced by the complexity of the learning materials and the instruction-induced load, respectively; Chen et al., 2023), and (5) the relationships between concurrently available components of information (e.g., the foreground to the background, the colorful to the black-and-white). The *individual-level processes* rely on (1) individual goals and meaning ascribed to given information, (2) cognitive processes such as stimulus-driven (bottom-up) and goal-directed (top-down) attention, working memory, and long-term memory, (3) germane cognitive load (the amount of cognitive resources, e.g., working memory, devoted to the task at hand; Korbach et al., 2018), (4) metacognitive processes, (5) previous experience, (6) affect, (7) motivation, and (8) attitudes. The *context of information relevance* comprises a myriad of features that are not embedded within the goal-driven activity, but nevertheless influence individual performance, such as (1) time constraints and (2) the sociocultural background of learners, teachers, and researchers. Of note, information relevance changes dynamically, that is, hinges not only on individual goals, but also on the outcomes of individual actions that preceded the present instance in which the individual is judging information relevance.

Numerous theoretical accounts of information relevance were conceived over the last century of psychological, educational, and computer science research, spanning decision making and judgment, attention and memory, critical information literacy, problem solving, and other. Some of these accounts, such as Cognitive Load Theory (Sweller, 2011), Self-Regulated Learning (Panadero, 2017), Leont'ev's Activity Theory (Leont'ev, 1979), or Cognitive-Affective Theory of Learning with Media (Moreno, 2005), guided the research presented in the Research Topic, partly overlapping with Figure 1. To our best knowledge, however, no theory has, to date, comprehensively accounted for all aspects of information relevance. Hopefully, the present Editorial and the Research Topic will inspire drafting and testing such novel, comprehensive theories that are yet to be developed.

## 3 Overview of the Research Topic

The present Research Topic comprises six empirical reports and a perspective article, covering diverse aspects of information relevance from the content features (Braunheim et al., Désiron and Schneider, Greeves and Oz, Lederer et al.) and the individual-level processes (Désiron and Schneider, Leclerq et al., Lederer et al., Vos et al.) to the context (Rhodes et al., Vos et al.). Several articles accounted for the real-world significance of information relevance judgments (Leclerq et al., Lederer et al., Rhodes et al., Vos et al.) and, specifically, their importance in online and multimedia environments (Braunheim et al., Désiron and Schneider, Greeves and Oz). Most articles focused on high school and college students, with only one involving 4- to 6-year-old preschoolers (Leclerq et al.).

Braunheim et al. investigated how young professionals at transition from educational to professional contexts utilize online information to achieve their goals. The study invoked Critical Online Reasoning skills and a theory of Metacognitive Activation to assess information relevance and reliability judgments in German beginner teachers, doctors, and lawyers during and after an open-ended web search. The authors found that the quality of arguments provided in the post-search assessment correlated with the quality of the selected sources.

Désiron and Schneider examined how high school and university students responded to colorful design when dealing with relevant information. The study built upon the Cognitive Load Theory, the Cognitive-Affective Theory of Learning with Media, and the Emotional Design Hypothesis to assess whether colorful design correlated with higher learning outcomes, and whether contrasting colors further lowered cognitive load. The results suggested that colorful designs indeed correlated with higher performance, and that color contrast lowered the participant-perceived extraneous but not the intrinsic cognitive load.

Greeves and Oz looked into differences in relevance judgments of YouTube videos between college instructors and students. Despite several similarities across groups, such as prioritizing video accuracy, content creators' expertise, or video duration, the students seemed to value additional features that would suggest community support for the content and the creator far more than the instructors.

Leclerq et al. employed analogical card sorting tasks to examine whether 4- to 6-year-old preschoolers could learn to use self-cueing strategies such as labeling and pointing to transfer rules across these tasks. In line with expectations, children trained on such strategies were more likely to spontaneously use them on the analogical task.

Lederer et al. assessed judging relevance of anecdotal, correlational, and experimental evidence in causal reasoning in preservice teachers and psychology students. Despite typical differences in methodological training across educational and psychological study programs, the authors found comparable performance levels across the two groups.

Rhodes et al. offered a new perspective on relevance of problem solving tasks by highlighting the importance of sociocultural factors on the researcher's and the participant's side. The authors recommended a checklist for researchers who wish to develop new problem solving tasks.

Vos et al. invoked their own conceptualization of information relevance as a function of object (“what?”), subject (“for whom?”), asserter (“according to whom?), and a purpose (“to what end?”) when examining the relevance of learning key mathematical concepts for high school students. Despite initial low levels of self-perceived relevance of such concepts, the students who participated in the study were shown to assert the relevance of the key concepts after learning about real-life applications and using their own imagination.

## 4 Limitations and outlook

The present Research Topic offers a broad outlook on information relevance judgments in educational and professional settings, but it suffered some limitations. Despite covering diverse aspects of such relevance, most articles emphasized the role of the content and the context rather than the individual-level processes. Furthermore, despite a broad age range covered in the Topic, from 4 (Leclerq et al.) to 46 (Braunheim et al.) years of age, all articles but one focused on high school and college students. None of the articles investigated information relevance judgments in dynamically changing information landscapes in aging populations. Finally, despite efforts at attracting authors from both the Global North and the Global South, all empirical articles published in the Research Topic involved sampling from Western, educated, industrialized, rich, and democratic (WEIRD; Rhodes et al.) societies. This may significantly limit the generalization of perspectives and results presented in the Research Topic beyond the Global Northern context.

Future research on information relevance should aim at developing comprehensive theoretical frameworks of information relevance, increasingly involve both young and aging participants, not only students and beginner professionals, and foster relevant collaborations beyond the WEIRD context.
